# Adventitious Shoot Regeneration from In Vitro Leaf Explants of the Peach Rootstock Hansen 536

**DOI:** 10.3390/plants9060755

**Published:** 2020-06-16

**Authors:** Angela Ricci, Luca Capriotti, Bruno Mezzetti, Oriano Navacchi, Silvia Sabbadini

**Affiliations:** 1 Department of Agricultural, Food and Environmental Sciences, Marche Polytechnic University, 60131 Ancona, Italy; angela.ricci@pm.univpm.it (A.R.); l.capriotti@pm.univpm.it (L.C.); b.mezzetti@staff.univpm.it (B.M.); 2 Vitroplant Italia, 47521 Cesena, Italy; orianonavacchi@vitroplant.it

**Keywords:** *Prunus persica*, in vitro organogenesis, somatic tissue, STS, carbenicillin, cefotaxime

## Abstract

In the present study, an efficient system for the in vitro regeneration of adventitious shoots from the peach rootstock Hansen 536 leaves has been established. Twenty regeneration media containing McCown Woody Plant Medium (WPM) as a basal salt supplemented with different concentrations and combinations of plant growth regulators (PGRs) were tested. Expanded leaves along with their petiole from 3-week-old elongated in vitro shoot cultures were used as starting explants. The highest regeneration rate (up to 53%) was obtained on WPM basal medium enriched with 15.5 μM N^6^-benzylaminopurine (BAP). The influences on leaf regeneration of the ethylene inhibitor silver thiosulphate (STS) and of different combinations of antibiotics added to the optimized regeneration medium were also investigated. The use of 10 μM STS or carbenicillin (238 μM) combined with cefotaxime (210 μM) significantly increased the average number of regenerating shoots per leaf compared to the control. In vitro shoots were finally elongated, rooted and successfully acclimatized in the greenhouse. The results achieved in this study advances the knowledge on factors affecting leaf organogenesis in *Prunus* spp., and the regeneration protocol described looks promising for the optimization of new genetic transformation procedures in Hansen 536 and other peach rootstocks and cultivars.

## 1. Introduction

The genetic improvement of woody fruit species, including peach, through biotechnological approaches is often limited by the lack of tissue culture systems that allow the delivery of desired traits in the plant host genome and by the subsequent regeneration of the selected transgenic lines. Thus, the establishment of efficient in vitro adventitious regeneration methods represents a prerequisite for the development and application of effective transformation procedures aimed to genetically improve perennial crops [1].

Among fruit trees, peach is recognized as one of the most recalcitrant species in terms of in vitro organogenesis [2], and only a few studies report the establishment of efficient and reproducible regeneration protocols starting from different explants of peach rootstocks and cultivars. Although the use of adult tissues is highly recommended to preserve desirable traits of peach selected clones [3], adventitious shoots regeneration starting from seed-derived material has been the main successful approach used in the past for this species [4,5,6,7,8]. However, peach adventitious shoot regeneration, avoiding the use of juvenile tissues, has also been recorded starting from meristematic tissues [2,3,9] and leaves [10,11,12,13]. In particular, Gentile et al. (2002) described the only study reporting in vitro shoot organogenesis from leaves of *Prunus persica* L. cultivars (from expanded leaves, from leaves of preconditioned shoot apices and from leaves of adventitious shoots) [10]. Other results showing adventitious shoot regeneration from leaves have been reported for the peach hybrid rootstocks Nemaguard (*P. persica* × *P. davidiana*) [11], Guardian® (BY520-9 peach seedling rootstock) [12] and Hansen 536 (*P. persica × P. amygdalus*) [13]. These reports show the difficulty in setting up a versatile and efficient protocol for peach leaf organogenesis, which often depends on the genotype selected, and it is highly influenced by the starting explant used, by the macro-/microelements and plant growth regulators (PGRs) composing the basal culture medium and by the exposure to different hours/weeks of darkness/light during explant in vitro cultures. Furthermore, several studies describe how the accumulation of ethylene during in vitro tissue culture can have a negative impact on shoot regeneration (reviewed by Biddington [14]). Silver thiosulfate (STS), an ethylene inhibitor [15], has been successfully utilized as the promoter of adventitious shoot regeneration from leaf tissues of *P. armenica* L. [16], *P. serotina* Ehrh. [17] and *P. domestica* L. [18]. The only report investigating the effect of STS on shoot regeneration from peach leaf explants is reported by San et al. [12], where a regeneration rate of 16% of the peach rootstock Guardian® is reported.

As reported by several authors, the development of efficient protocols for shoot regeneration starting from somatic tissues of peach rootstocks is one of the primary objectives required for the application of gene-delivery technologies to this recalcitrant fruit crop [11,12,13,19]. In fact, peach rootstocks, such as peach × almond hybrids, have been extensively used for the propagation of peach plants, given their favourable agronomic traits, such as high tolerance to calcareous and dry soils, good productivity induced in the grafted peach scion, and resistance to several plant pathogens and pests [19,20,21]. Among peach × almond hybrid rootstocks, Hansen 536 (*P. persica* × *P. amygdalus*) has been used for grafting both peach and almond genotypes and has reached a significant commercial importance especially in California [13]. It is characterized by a high plant vigour also in poor and alkaline soils and shows resistance against some root-knot nematodes (*M. arenaria*, *M. incognita* and *M. javanica*) [19,22,23]. However, this genotype needs further genetic improvement especially due to its low waterlogging tolerance, which prevents its spread also in other geographical area [13,19].

In a recent paper, we described the optimization of an in vitro regeneration and transformation protocol for the peach hybrid rootstock Hansen 536 (*P. persica* × *P. amygdalus*) using meristematic bulks as starting explants [3]. Even though the regeneration rate obtained through this system was about 80%, only transgenic callus lines were obtained that did not lead to the regeneration of any transgenic plant. One possible solution to this bottleneck can be the development and use of a different regeneration protocol from somatic tissues, more suitable to obtain Hansen 536 transgenic plants in future transformation trials. The present study aimed to establish the optimal conditions necessary to induce an efficient organogenetic response from in vitro Hansen 536 leaf explants by evaluating the effect of different PGRs concentrations and combinations added to the basal regeneration medium. Furthermore, the impact of different compounds that could significantly enhance adventitious shoot regeneration efficiency was evaluated. In particular, we studied the influence of STS on Hansen 536 shoot regeneration as well as the effect on the regeneration efficiency of different antibiotics, which are often used in genetic transformation systems to contain *Agrobacterium* overgrowth. The established protocol represents a useful tool for the optimization of new genetic transformation methods and for the application of biotechnological approaches aimed at improving genetically Hansen 536 and other peach rootstocks and varieties.

## 2. Results

### 2.1. Influence of Different Combinations and Concentrations of PGRs on Regeneration Efficiency from Leaf Explants

Twenty regeneration media (WPM 1–WPM 20) supplemented with different concentrations and combinations of PGRs were tested to evaluate the organogenetic competence and adventitious shoot regeneration efficiency of Hansen 536 leaf explants (Table 1). Three weeks after incubation in dark condition, calli appeared on petioles and formed around the cuts made perpendicular to the leaf mid vein, and some buds and adventitious shoots started to regenerate, mainly on the petiole surface. After five weeks of culture, about 100% caulogenesis was observed on leaves cultured on most of the tested regeneration media (except for WPM 1, where no caulogenesis was recorded) (data not shown). Differences were observed for callus size based on the PGR combination used (Supplementary Figure S1). Data acquired after five weeks from the beginning of the trial revealed higher values of both regeneration frequency and average number of regenerating shoots per leaf when the explants were cultured on basal medium supplemented with different concentrations of N^6^-benzylaminopurine (BAP) used alone or in combination with 0.25 μM α-naphtalene acetic acid (NAA) (Table 1). In particular, the highest regeneration frequency and mean number of regenerating shoots per leaf, 53% and 0.77 ± 0.08, respectively, were obtained from explants cultured on medium containing 15.5 μM BAP alone (WPM 11), whereas the analysis of results obtained by using different cytokinins other than BAP in the basal medium, like kinetin (KIN) or thidiazuron (TDZ), showed a significant decrease in the organogenetic response of leaf explants, with regeneration rates up to 13% and 28% for WPM 6 and WPM 15, respectively. Furthermore, the combined use of NAA together with TDZ in the media seemed to have a negative influence on adventitious shoot regeneration compared to the use of TDZ alone (Table 1).

### 2.2. Influence of Silver Thiosulphate on Regeneration Efficiency from Leaf Explants

**Figure 1:**
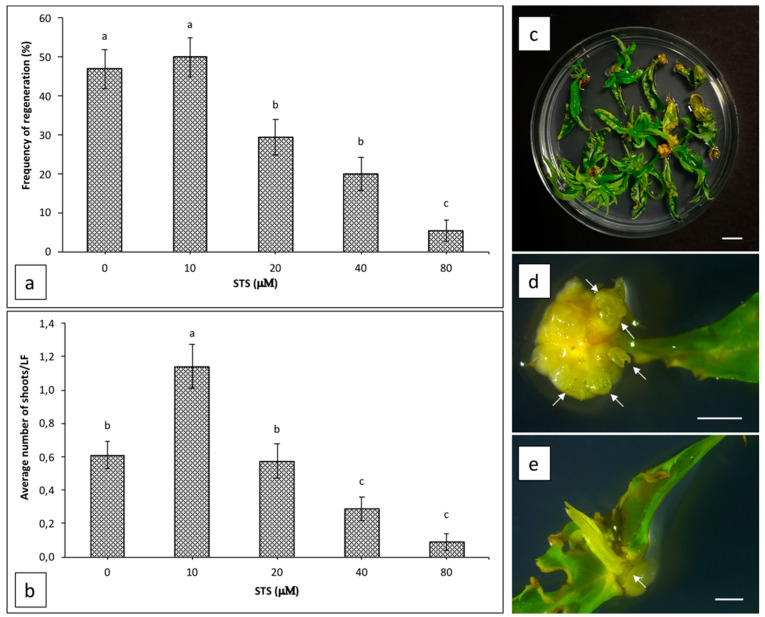
Regeneration efficiency of Hansen 536 leaf explants cultured on WPM 11 supplemented with different concentrations of silver thiosulphate (STS): (**a**) frequency of regeneration expressed as the number of explants regenerating at least one shoot per total explants treated × 100 and (**b**) the average number of regenerating shoots per leaf (LF) after five weeks of culture. One-way ANOVA was used to analyse the results. Different letters show significant differences at *p* < 0.05 by the Newman–Keuls test ± SE (*n* = 150). Each value represents the mean ± SE of three independent experiments. (**c**) Adventitious shoots regenerating from leaf explants after seven weeks of culture on WPM 11 supplemented with 10 μM STS (bar = 1 cm). Adventitious shoots regenerating from petiole (**d**) and from cuts perpendicular to the leaf mid vein (**e**) of Hansen 536 leaves after three weeks of culture on WPM 11 supplemented with 10 μM STS (bar = 2 mm). White arrows indicate regenerating adventitious shoots.

The effect of silver thiosulphate (STS) supplemented in WPM 11 medium on shoot organogenesis from Hansen 536 leaf explants was evaluated after five weeks of culture (Figure 1a,b). STS did not increase explant regeneration frequency at any of the concentrations tested. The highest value (50%) was obtained by adding 10 μM STS to the regeneration medium, and it was not significantly different to the regeneration frequency observed from leaves placed on regeneration medium without STS (47%) (Figure 1a), whereas the average number of regenerating shoots per leaf increased two-fold (a mean of 1.14 ± 0.13 shoots per explant) when STS was used at a concentration of 10 μM compared to the control (a mean of 0.61 ± 0.08 shoots per explant) (Figure 1b–d). Concentrations of STS higher than 10 μM inhibited adventitious shoot regeneration progressively; in particular, when STS was used at 20, 40 or 80 μM, the regeneration frequency decreased drastically to 29.3%, 20% and 5.3%, respectively (Figure 1a), while the average number of regenerating shoots per leaf decreased significantly by using a concentration of STS equal or higher than 40 μM (Figure 1b). Furthermore, when STS was added to the regeneration medium at any concentration tested, we observed a higher frequency of shoots that also regenerated on the cuts made perpendicular to the leaf mid vein (Figure 1e), while most of the shoots regenerated only on petiole surface when explants were cultured without STS (data not shown).

### 2.3. Influence of Antibiotics on Regeneration Efficiency from Leaf Explants

The effect of different antibiotics added to WPM 11 medium on adventitious shoot regeneration efficiency of Hansen 536 leaves was observed after five weeks of culture (Table 2). Similar to the results obtained when STS was supplemented in the basal regeneration medium, the addition of antibiotics also did not significantly increase the regeneration frequency of leaf explants compared to the control. However, the addition of carbenicillin (238 μm) plus cefotaxime (210 μm) in the WPM 11 medium positively affected the average number of regenerating shoots per leaf compared to the control and to the other antibiotics tested (Table 2). On the contrary, when the same antibiotics were applied alone at higher concentrations, regeneration rates decreased more than two-fold.

### 2.4. In Vitro Rooting and Acclimatization of Adventitious Shoots

**Figure 2:**
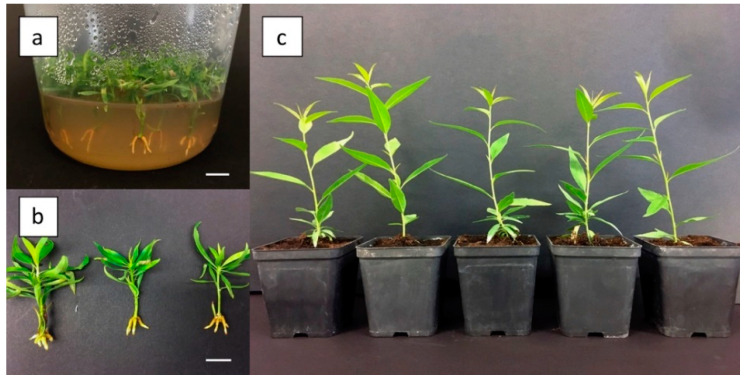
In vitro rooting and acclimatization of adventitious shoots from Hansen 536 leaves: (**a**,**b**) elongated and rooted in vitro adventitious shoots ready for acclimatization and (**c**) acclimatized rooted shoots in 7 × 7 cm pots.

Adventitious shoots developed in the above treatments were in vitro elongated, rooted, and acclimatized. About 85% of them were able to produce in vitro roots (about 0.5–1 cm long) after 20 days in the rooting medium (Figure 2a,b), and about 90% of them were successfully acclimatized to the greenhouse (Figure 2c).

## 3. Discussion

Efficient adventitious shoot regeneration, rooting, and acclimatization were achieved in this study by using leaves of the peach × almond hybrid rootstock Hansen 536 as the starting plant material. One of the most important factors affecting plant tissue regeneration is the type and concentrations of PGRs added to the regeneration medium. In this study, the highest regeneration efficiency (53%, with mean number of shoots per explant of 0.77 ± 0.08) was obtained when WPM basal medium was enriched with 15.5 μM BAP (WPM 11), albeit good regeneration rates (from 29.3% up to 37%) were also obtained when explants were placed on media supplemented with BAP in combination with NAA. Similarly, Gentile et al. [10], reporting in vitro shoot induction from *P. persica* leaves, showed that the best results were obtained from leaves when BAP was used as cytokinin in the media; the highest shoot regeneration rate of 28.3% was recorded in peach cv. 842 Standard. Furthermore, Zong et al. [13] have recently optimized a protocol for Hansen 536 in vitro leaf regeneration, which led to a maximum shoot regeneration rate of 36.1% when explants were cultured on WPM media supplemented with BAP in combination with indole-3-butyric acid IBA, confirming the positive influence conferred by the use of BAP as a cytokinin on leaf organogenesis from peach. However, other studies have reported better results on peach leaf regeneration when cytokinins other than BAP were added to the culture medium. As observed by Zhou et al. [11], leaves of the peach rootstock Nemaguard induced the best regeneration of shoots (71.7%, with mean number of shoots of 5.74 ± 3.24) when cultured in the presence of TDZ as cytokinin (9.08 μM TDZ + 0.54 μM IBA). Additionally, San et al. [12] observed a positive effect conferred by the use of TDZ instead of BAP as a cytokinin for shoot regeneration from Guardian^®^ in vitro leaves. However, in this study, the presence of TDZ combined with NAA was not sufficient to induce shoot organogenesis, but the addition of the ethylene inhibitor STS at a concentration of 10 μM was an essential factor to obtain an organogenetic response (regeneration rate of 16% with a mean number of shoots of 1.6). Our results showed that the use of TDZ induced a significant reduction in the regeneration of shoots from Hansen 536 leaves compared to WPM 11 medium, especially when TDZ was used in combination with NAA in the regeneration media. Similarly, in our study, the regeneration rates obtained by the use of KIN as a cytokinin were significantly lower (13.3% of regeneration) compared to the use of BAP alone; however, to our knowledge, this represents the first successful attempt to induce adventitious regeneration from peach leaves through the use of KIN. Indeed, other authors reported the effect of KIN on caulogenesis from peach leaves, which did not lead to the regeneration of any adventitious shoot [24,25]. Although all the studies mentioned above underline the difficulty in obtaining a versatile protocol to be applied for leaf organogenesis in different peach genotypes, it seems that the presence of the petiole as part of the starting leaf explant represents a critical factor for a successful adventitious shoot regeneration from leaves of peach rootstocks and cultivars, as also reported by Gentile et al. [10] and by other authors in different *Prunus* species [17,25,26]. Indeed, in all these studies, including ours, most of the regenerated adventitious shoots were observed at the petiole. Several studies carried out on *Prunus* spp. have also demonstrated that the interaction among different factors, other than the medium composition, concur in the organogenetic response of in vitro leaf explants, such as plant source conditions, the age of explant, cut side and leaf orientation on the culture substrate [11,27]. In our study, we observed a very low frequency of leaf organogenesis when Hansen 536 explants were cultured with the adaxial surface in contact with WPM11 medium (Supplementary Figure S2; data not shown) in contrast with the results obtained by Zong et al. on the same genotype [13] and by Gentile et al. on peach cultivars [10]. Several authors have examined the influence of leaf orientation on explant regeneration frequency in different plant species, however without obtaining a common response [27,28,29,30,31,32]. In general, it seems that a higher regeneration efficiency should be achieved when the wounded surface of the leaf is in contact with the medium, probably due to a better absorption of nutrients and PGRs from the substrate [27,33]. Furthermore, the different organogenetic responses of the leaf in its two different orientations can be connected to the total surface area that is in contact with the substrate. Positioning the abaxial side of the explant on the medium avoids the typical curling of both ends of the leaf that occurs when it is oriented with the adaxial surface on the substrate [28,31].

In the present study, we also investigated the influence of different concentrations of STS supplemented in the culture medium on the regeneration frequency and mean number of shoots from Hansen 536 leaves. Different authors stated that STS has a positive role in shoot organogenesis from leaf tissues of *Prunus* spp., including peach [12,16,18]. This effect seems to be associated to the action of Ag^+^ ions present in this compound, which block ethylene signal transduction induced by in vitro cultured explants [16]. The ability of Ag^+^ ions to inhibit ethylene action seems to prevent some of its negative effect during shoots organogenesis [34,35]. In our study, we observed a significant increase in the average number of shoots per explant when STS was used at a concentration of 10 μM. Furthermore, the effect of this ethylene inhibitor on Hansen 536 leaf organogenesis was dose-dependent; in particular, when STS was used at concentrations higher than 10 μM, adventitious shoot regeneration was progressively inhibited. Our results together with previously published data reported by San and collaborators [12] suggest 10 μM as the optimal concentration of STS capable of improving the adventitious regeneration of peach leaves. Other studies have already reported that STS and similar compounds, like silver nitrate, are biologically active at very low concentrations for some plant species [34,36]; in these cases, if the maximum effective concentration of STS is reached, toxic effects on plant tissues are induced and regeneration responses are inhibited, as also observed in our study. This also suggests that, at the right level, the presence of ethylene in the tissue culture is still needed for an efficient organogenetic response, as also reported by other studies [37,38]. Thus, as remarked by Petri and Scorza [18], the optimum STS concentration in *Prunus* spp. and other plant species is often genotype-dependent, making essential the evaluation of STS impact on shoot regeneration from each cultivar or rootstock.

Lastly, the influence of different antibiotics included in the WPM 11 medium on the regeneration efficiency from Hansen 536 leaves was evaluated; this trial aimed to test only the antibiotics commonly used during *Agrobacterium*-mediated transformation trials to avoid bacterial persistence in the medium post-transformation. Several studies reported the positive effect of antibiotics on organogenetic processes in different plant species included *Prunus* spp. [16,39,40,41,42,43,44,45,46,47,48,49,50,51]. We observed that carbenicillin (238 μM) combined with cefotaxime (210 μM) improved the average number of shoots per explant compared to the control, whereas the same antibiotics used alone at higher concentrations decreased regeneration efficiency (Table 2). Carbenicillin has been often tested to improve plant organogenesis, and it seems that the positive effect reported by different studies can be related to the byproducts released during its breakdown, which show an auxin-like activity with beneficial effects on shoot development in various plant species [16,39,44,45,46,47,48]. In our study, a concentration of carbenicillin higher than 238 μM has probably induced an excess in auxin activity in peach leaf tissues, which led to a considerable reduction of regeneration frequency from Hansen 536 leaves. Similarly, a toxic effect of this antibiotic used at concentrations higher than 238 μM was also observed by other authors working with different plant species other than *Prunus* spp. [49,52,53]. Differently from carbenicillin, the chemical conformation of cefotaxime does not suggest the production of auxin-like compounds as byproducts during its breakdown [48,54]; thus, additional studies are needed to understand the mode of action of this antibiotic and its involvement in plant metabolism. Our results show that the use of cefotaxime at concentrations higher than 210 μM leads to a reduction in regeneration efficiency; the negative effect of cefotaxime on plant organogenesis has also been reported in other studies [48,52,55], even though the neutral or beneficial impacts of this antibiotic on morphogenesis of several plant species [39,40,41,42,46,49,50,51,56], including *Prunus* spp. [16], have been the main outcome observed until date.

In conclusion, we set up a valid and reproducible adventitious shoot regeneration system from Hansen 536 leaf tissues with a frequency up to 53% when explants were cultured on WPM medium enriched with 15.5 μM BAP. The established protocol has improved the regeneration efficiency of Hansen 536 leaves compared with maximum results reported by Zong et al. [13] (regeneration frequency up to 36%) on the same genotype, probably due to the interaction of different factors, such as different sources of starting plant material, medium composition, leaf orientation on the substrate and type of leaf incision. Furthermore, we identified the optimal concentration of STS which significantly improved shoot regeneration by increasing two-fold the average number of shoots per explant. In addition, the regeneration medium enriched with carbenicillin combined with cefotaxime at the concentration tested induced a significant increase of regenerating shoots per leaf. This result has particular relevance for the genetic transformation of this peach genotype. In fact, these kinds of antibiotics are normally used during genetic transformation processes to contain *Agrobacterium* persistence in the medium after transforming plant tissues; therefore, it is essential to avoid negative effects on regeneration efficiency that could be caused by these compounds [16]. The overall results obtained in this study should improve the knowledge on factors controlling peach leaf organogenesis, and the results achieved by the best combination of the tested factors look promising for the optimization of new genetic transformation protocols in Hansen 536 and other peach rootstocks and cultivars.

## 4. Materials and Methods

### 4.1. Establishment of In Vitro Shoots

Shoot tips (approximately 0.5 cm long) were cut from 10-cm-long shoots of 10-year-old greenhouse-grown peach × almond hybrid rootstock Hansen 536 (*P. persica* × *P. amygdalus*) trees at Vitroplant Italia, Cesena, Italy and used to establish peach in vitro cultures. The collected shoot tips were surface sterilized by washing them in 1% (V/V) sodium hypochlorite solution for 15 min, followed by three washes with sterile distilled water. Explants were then placed on shoot multiplication medium composed of McCown Woody Plant Medium (WPM) (Duchefa Biochemie, Haarlem, The Netherlands) basal salts and vitamins [57], 30 g L^−1^ sucrose and 5 g L^−1^ plant agar S1000 (B&V, Reggio Emilia, Italy) [3] supplemented with 6.6 μM N^6^-benzylaminopurine (BAP) (Duchefa Biochemie, Haarlem, The Netherlands) and 0.1 μM α-naphtalene acetic acid (NAA) (Duchefa Biochemie, Haarlem, The Netherlands). The final pH value was adjusted to 5.7 with KOH before autoclaving at 121 °C for 20 min. In vitro cultures were kept in a growth chamber at 24 ± 1 °C under a photoperiod of 16-h light (70 μmol/m^2^/s) provided by white fluorescent tubes, and they were periodically subcultured (2-week intervals) on fresh shoot multiplication medium for a total of three subcultures. Proliferating shoots were then transferred to the elongation medium in order to provide expanded leaves for this study. Preparation and composition of the elongation medium were the same as for the shoot multiplication medium, except for plant growth regulators (PGRs) concentrations, corresponding to 0.45 μM BAP and 0.1 μM NAA. In vitro shoots were maintained on the elongation medium for 20 days at the same light and temperature conditions as described above and then used for shoot regeneration trials.

### 4.2. General Approach for Adventitious Shoot Regeneration

**Figure 3:**
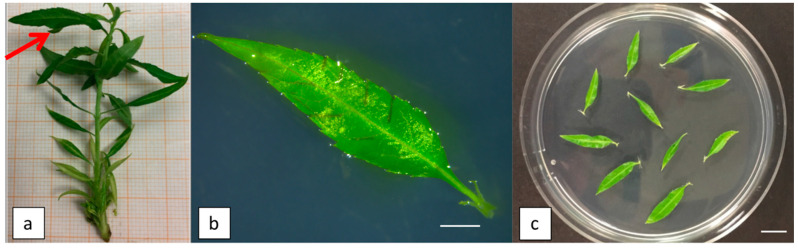
Plant material used as the starting explants in this study: (**a**) leaves collected from 3-week-old elongated shoots of Hansen 536. The arrow indicates the type of leaf collected and used in all the regeneration experiments; (**b**) the young leaf of the Hansen 536 wounded perpendicular to the mid vein and placed with the abaxial side on regeneration medium (bar = 2 mm), and (**c**) in vitro leaves of Hansen 536 placed on regeneration medium (bar = 1 cm).

The first four apical expanding leaves (about 1.5–2 cm in length) along with their petiole from 3-week-old elongated shoot cultures were used as starting explants in this study (Figure 3a). The abaxial surface of each leaf was wounded about four times at each side perpendicular to the leaf mid vein, leaving the sections intact, and then placed with the abaxial side in contact with the regeneration medium (Figure 3b,c). Leaves were cultured on WPM basal salts and vitamins, 30 g L^−1^ sucrose and 5 g L^−1^ plant agar S1000 (B&V, Reggio Emilia, Italy); the pH was adjusted to 5.7 before autoclaving at 121 °C for 20 min, and then 25 mL of medium was poured into sterile plastic Petri dishes (9 cm × 1.5 cm). After leaves were placed on medium, the dishes were maintained in darkness at 24 ± 1 °C for three weeks. In vitro leaves were then transferred to fresh media and exposed to light (16-h photoperiod at a light intensity of 40 μmol/m^2^/s) at 24 ± 1 °C. Data on the leaf regeneration frequency and on the average number of regenerating shoots per leaf were collected after five weeks from the beginning of the trial.

### 4.3. Effect of Different Combinations and Concentrations of PGRs

In this experiment, leaves were placed on regeneration media supplemented with cytokinins, as kinetin (KIN) (Duchefa Biochemie, Haarlem, The Netherlands) (14, 16.3 or 19 μM), BAP (11, 13.3 or 15.5 μM) or thidiazuron (TDZ) (Duchefa Biochemie, Haarlem, The Netherlands) (2.25, 4.5 or 9 μM) and with auxin, as NAA (0.25 μM), for a total of 20 different media combinations (Table 3).

### 4.4. Effect of Silver Thiosulphate

Young expanding leaves were placed on WPM 11 regeneration medium supplemented with different concentrations of silver thiosulphate (STS) (0, 10, 20, 40 or 80 μM). Preparation of media and culture conditions of the leaves were the same as previously described.

Stock solutions (0.1 M) of sodium thiosulphate and silver nitrate were prepared by dissolving 790 mg of sodium thiosulphate (Sigma-Aldrich, Milan, Italy) and 850 mg of silver nitrate (Sigma-Aldrich, Milan, Italy) into 50 mL of ultrapure water. STS stock solution (0.02 M) was then prepared just before use by slowly adding 20 mL of silver nitrate stock solution (0.1 M) into 80 mL of sodium thiosulphate stock solution (0.1 M) (1:4 ratio). STS stock solution was filter-sterilised and added to the medium after autoclaving and cooling down to 50 °C.

### 4.5. Effect of Antibiotics

The influence of different antibiotics added to the WPM 11 medium, alone or combined (Table 2), on the regeneration of Hansen 536 leaves was evaluated using the same regeneration protocol previously reported. Filter-sterilised antibiotics were added to the regeneration medium after autoclaving and cooling down to 50 °C.

### 4.6. Shoots Elongation, Rooting and Acclimatization

Shoots (1 cm in length) regenerated from leaf explants in the above experiments were excised and propagated on WPM medium, 30 g L^−1^ sucrose and 5 g L^−1^ plant agar S1000 (B&V, Reggio Emilia, Italy) supplemented with 6.6 μM BAP and 0.1 μM NAA for a total of three subcultures (2-week intervals). Single shoots were then placed on WPM medium supplemented with 0.5 μM BAP and 7.38 μM indole-3-butyric acid (IBA) (Duchefa Biochemie, Haarlem, The Netherlands) for 20 days to induce rooting. Shoot cultures were placed in the growth chamber under a photoperiod of 16-h light (70 μmol/m^2^/s) provided by white fluorescent tubes. In vitro rooted shoots were finally acclimatized in pots (7 × 7 cm) containing commercial peat and grown in the greenhouse.

### 4.7. Statistical Analysis

For each treatment included in the regeneration experiments described above, five petri dishes were prepared and a total of fifty explants were used (ten leaves per dish). Three independent experiments were carried out for each regeneration trial. Regeneration frequency is expressed as (number of explants regenerating at least one shoot/total explants treated) × 100. Data on percent regeneration were transformed by the arcsine square root transformation, ARSIN (SQRT (X)), before analysis. Shoot numbers are shown as the mean ± SE of the total number of shoots regenerating from starting leaves.

The results acquired were analyzed by one-way ANOVA using Statistica 7 software (Statsoft Tulsa, CA, USA), and means were separated using the Newman–Keuls test (*p* < 0.05).

## Figures and Tables

**Table 1:** Influence of plant growth regulators (PGRs) on the regeneration efficiency from Hansen 536 leaf (LF) explants cultured for five weeks on regeneration media.

Regeneration Media	Frequency of Regeneration (%) ± SE *	Average Number of Regenerating Shoots/LF ± SE
WPM 1	0 ^(g)^	0 ^(e)^
WPM 2	0 ^(g)^	0 ^(e)^
WPM 3	8.7 ± 2.3 ^(fg)^	0.12 ± 0.03 ^(de)^
WPM 4	9.3 ± 2.38 ^(fg)^	0.11 ± 0.04 ^(de)^
WPM 5	6.7 ± 2.04 ^(fg)^	0.07 ± 0.02 ^(de)^
WPM 6	13.3 ± 2.78 ^(defg)^	0.15 ± 0.03 ^(cde)^
WPM 7	3.3 ± 1.47 ^(g)^	0.03 ± 0.0.1 ^(e)^
WPM 8	2 ± 1.15 ^(g)^	0.02 ± 0.01 ^(e)^
WPM 9	26.7 ± 3.62 ^(bcd)^	0.41 ± 0.06 ^(b)^
WPM 10	24.7 ± 3.53 ^(bcde)^	0.39 ± 0.06 ^(b)^
WPM 11	53 ± 4.02 ^(a)^	0.77 ± 0.08 ^(a)^
WPM 12	29.3 ± 3.73 ^(bc)^	0.47 ± 0.07 ^(b)^
WPM 13	32 ± 3.82 ^(bc)^	0.39 ± 0.05 ^(b)^
WPM 14	37 ± 3.84 ^(b)^	0.48 ± 0.06 ^(b)^
WPM 15	28 ± 3.88 ^(bc)^	0.48 ± 0.06 ^(b)^
WPM 16	20 ± 3.59 ^(cdef)^	0.3 ± 0.06 ^(bcd)^
WPM 17	24 ± 3.49 ^(bcde)^	0.36 ± 0.07 ^(bc)^
WPM 18	12 ± 3.19 ^(efg)^	0.16 ± 0.05 ^(de)^
WPM 19	2 ± 2.72 ^(g)^	0.02 ± 0.04 ^(e)^
WPM 20	6 ± 2.04 ^(fg)^	0.06 ± 0.03 ^(de)^

* Number of explants regenerating at least one shoot per total explants treated × 100. One-way ANOVA was used to analyse the results. Different letters in the same column show significant differences at *p* < 0.05 by the Newman–Keuls test ± SE (*n* = 150). Each value represents the mean ± SE of three independent experiments. WPM, McCown Woody Plant Medium.

**Table 2:** Effect of antibiotics on the regeneration efficiency from Hansen 536 leaf (LF) explants cultured for five weeks on WPM 11 medium.

Antibiotic(s) ^w^	μM ^y^	Frequency of Regeneration (%) ± SE ^z^	Average Number of Regenerating Shoots/LF ± SE
None	0	49 ± 5.02 ^(a)^	0.61 ± 0.08 ^(b)^
Carbenicillin	475	19.3 ± 3.68 ^(b)^	0.4 ± 0.07 ^(bc)^
Cefotaxime	420	21.4 ± 3.86 ^(b)^	0.33 ± 0.06 ^(bc)^
Timentin ^x^	514	9.3 ± 1.41 ^(b)^	0.17 ± 0.01 ^(c)^
Carbenicillin/Cefotaxime	238/210	43 ± 4.94 ^(a)^	0.91 ± 0.13 ^(a)^
Timentin/Cefotaxime	171/210	21.3 ± 3.94 ^(b)^	0.39 ± 0.07 ^(bc)^

^w^ Duchefa Biochemie, Haarlem, The Netherlands. ^x^ 15:1 mixture of ticarcillin and clavulanic acid. ^y^ Common concentrations found in the literature to control *Agrobacterium* growth. ^z^ Number of explants regenerating at least one shoot per total explants treated × 100. One-way ANOVA was used to analyse the results. Different letters in the same column show significant differences at *p* < 0.05 by the Newman–Keuls test ± SE (*n* = 150). Each value represents the mean ± SE of three independent experiments.

**Table 3:** Different concentrations and combinations of PGRs used in the regeneration medium.

	Plant Growth Regulators (μM)			
	KIN	BAP	TDZ	NAA
WPM 1	-	-	-	-
WPM 2	-	-	-	0.25
WPM 3	14	-	-	-
WPM 4	16.3	-	-	-
WPM 5	19	-	-	-
WPM 6	14	-	-	0.25
WPM 7	16.3	-	-	0.25
WPM 8	19	-	-	0.25
WPM 9	-	11	-	-
WPM 10	-	13.3	-	-
WPM 11	-	15.5	-	-
WPM 12 WPM 13 WPM 14 WPM 15 WPM 16 WPM 17 WPM 18	- - - - - - -	11 13.3 15.5 - - - -	- - - 2.25 4.5 9 2.25	0.25 0.25 0.25 - - - 0.25
WPM 19	-	-	4.5	0.25
WPM 20	-	-	9	0.25

KIN, kinetin; BAP, N^6^-benzylaminopurine; TDZ, thidiazuron; NAA, α-naphtalene acetic acid.

## Supplementary Materials

The following are available online at https://www.mdpi.com/2223-7747/9/6/755/s1, Figure S1: Caulogenesis and leaf regeneration from Hansen 536 explants. Figure S2: Hansen 536 leaf explants cultured with the abaxial or adaxial sides on regeneration medium.
