# Supplementary material for: Adventitious Shoot Regeneration from In Vitro Leaf Explants of the Peach Rootstock Hansen 536
Source: Plants (Basel). 2020 Jun 16;9(6):755. doi: 10.3390/plants9060755 (PMC7357001; doi:10.3390/plants9060755)
Supplement: Supplementary file 1 [file plants-09-00755-s001.pdf]

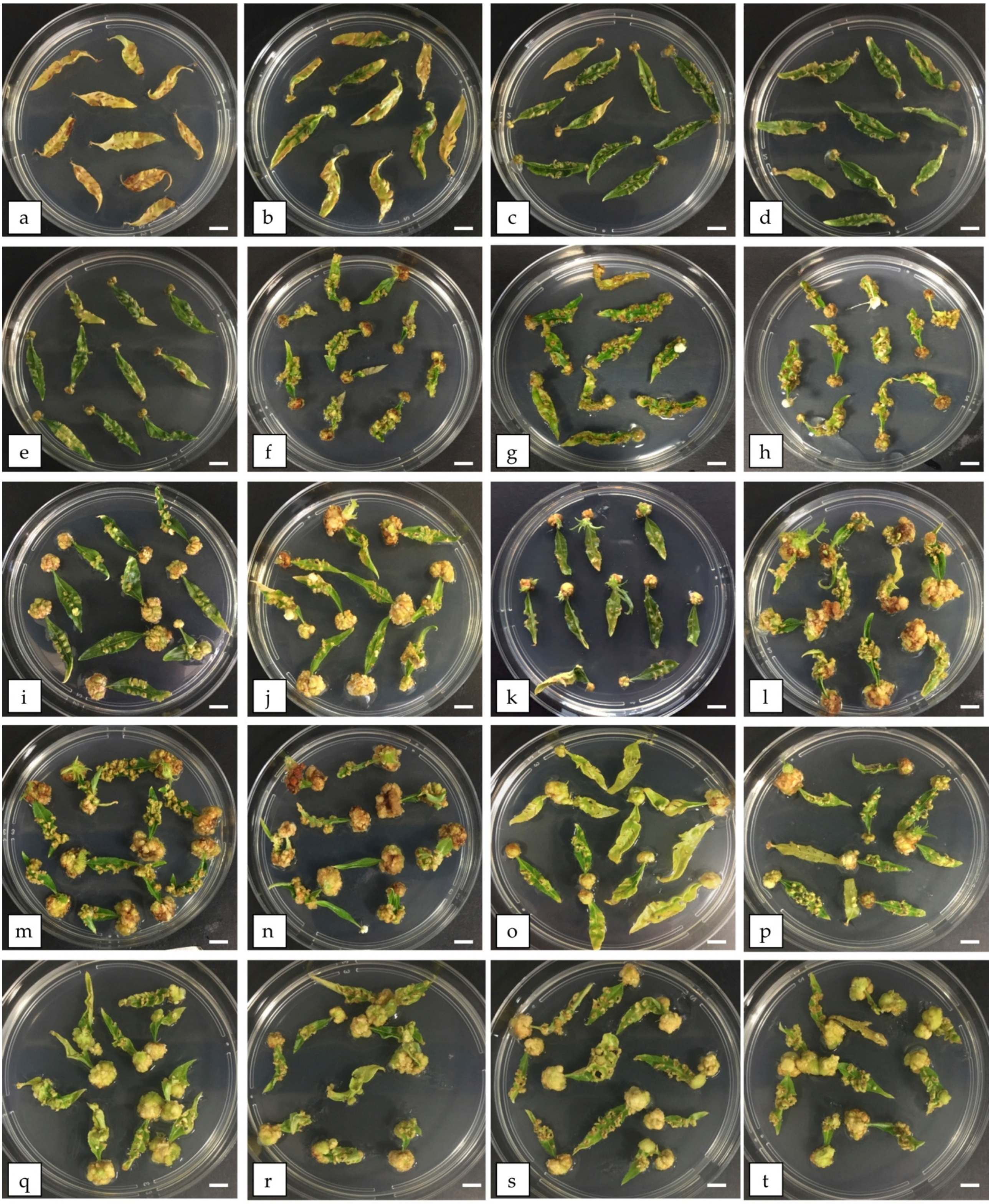

Supplementary figure 1. Caulogenesis and leaf regeneration from Hansen 536 explants placed on WPM 1 - WPM 20 **(a - t)** media after five weeks from the beginning of the experiment (bar = 1 cm).

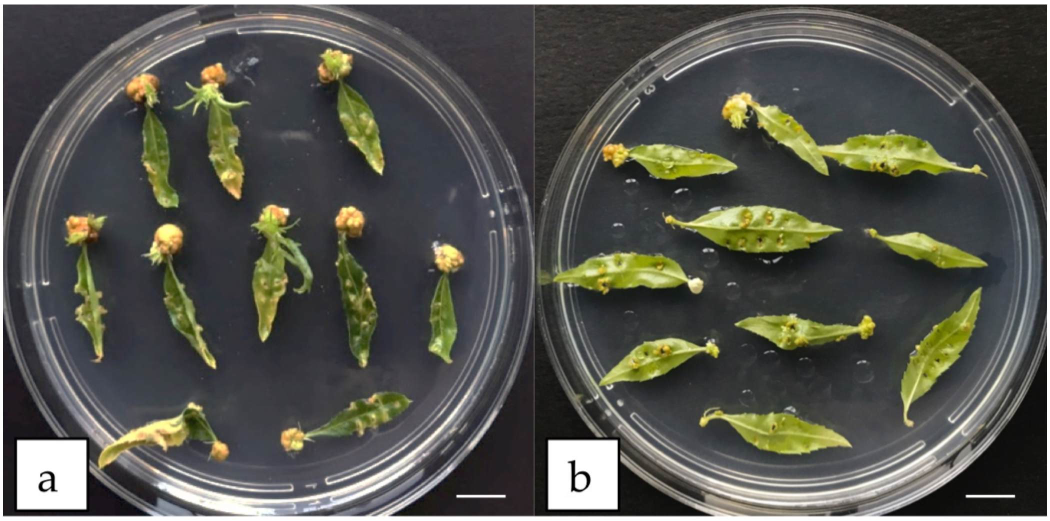

Supplementary figure 2. Hansen 536 leaf explants after five weeks of culture with the abaxial side **(a)** or the adaxial side **(b)** on WPM11 medium (bar = 1 cm).
